# Investigation of methicillin-resistant *Staphylococcus aureus*, methicillin-susceptible *Staphylococcus aureus*, and *Staphylococcus argenteus* from wild long-tailed macaques (*Macaca fascicularis*) at Kosumpee Forest Park, Maha Sarakham, Thailand

**DOI:** 10.14202/vetworld.2022.2693-2698

**Published:** 2022-11-26

**Authors:** Natapol Pumipuntu, Thanyaphorn Chamnandee, Kittisak Saengthong, Suvit Pathomthanasarn, Tawatchai Tanee, Pensri Kyes, Penkhae Thamsenanupap, Apichat Karaket, Marilyn C. Roberts, Randall C. Kyes

**Affiliations:** 1 One Health Research Unit, Mahasarakham University, Maha Sarakham, Thailand; 2 Veterinary Infectious Disease Research Unit, Mahasarakham University, Maha Sarakham, Thailand; 3 Faculty of Veterinary Sciences, Mahasarakham University, Maha Sarakham, Thailand; 4 Faculty of Environment and Resource Studies, Mahasarakham University, Maha Sarakham, Thailand; 5 Department of Psychology, Center for Global Field Study, and Washington National Primate Research Center, University of Washington, Seattle, Washington, USA; 6 Department of National Parks, Wildlife and Plant Conservation, Bangkok, Thailand; 7 Department of Environmental and Occupational Health, University of Washington, Seattle, Washington, USA; 8 Departments of Psychology, Global Health, and Anthropology, Center for Global Field Study, and Washington National Primate Research Center, University of Washington, Seattle, Washington, USA

**Keywords:** *Macaca fascicularis*, methicillin-resistant *Staphylococcus* *aureus*, methicillin-susceptible *Staphylococcus aureus*, non-ribosomal peptide synthetase gene, *Staphylococcus argenteus*

## Abstract

**Background and Aim:**

In the past, the prevalence of methicillin-resistant *Staphylococcus* *aureus* (MRSA) infections in both humans and animals has increased across Thailand. *Staphylococcus argenteus* has been associated with infections among humans, exotic pets, and livestock. Both species have been identified in non-human primate species from geographically diverse locations but not from non-human primates in Thailand. This study aimed to determine the presence of MRSA/methicillin-susceptible *S. aureus* (MSSA) and *S. argenteus* isolates collected from buccal swab samples in *Macaca fascicularis* at Kosumpee Forest Park (KFP), Maha Sarakham, Northeast Thailand.

**Materials and Methods:**

Aseptic buccal swab samples were collected from 30 free-ranging macaques in November 2018. All isolates were tested using multiple biochemical tests and *S. aureus* latex slide agglutination test. Presumptive *S. aureus* isolates were tested for the presence of the *mecA* gene using polymerase chain reaction (PCR) assays. The isolates were phenotypically determined to be resistant to a β-lactam antibiotic using the disk diffusion method with a 30 mg cefoxitin disk. The isolates were analyzed by PCR for the non-ribosomal peptide synthetase (*NRPS*) gene to distinguish *S. argenteus* from *S. aureus*.

**Results:**

Fifteen macaques (50%) were colonized with *S. aureus* and 21 isolates were characterized. Three of the macaques carried both the MRSA and MSSA isolate. One animal carried both MRSA and *S. argenteus* isolate, and one animal carried only *S. argenteus*. The *NRPS* gene analysis confirmed that 2 isolates (9.52%) were *S. argenteus* and 19 isolates (90.48%) were *S. aureus* [five MSSA and 14 MRSA].

**Conclusion:**

This study is the first to identify MRSA/MSSA and *S. argenteus* in wild free-ranging *M. fascicularis* from Thailand at the KFP in Maha Sarakham. This study is also the first report on the occurrence of *S. argenteus* carriage in *M. fascicularis* from Thailand.

## Introduction

Thailand’s long-tailed macaques (*Macaca fascicularis*) are the most frequently encountered non-human primate species in the country [1]. These macaques are well adapted to living in close proximity to humans and are widely distributed in urban areas [2–5]. As such, interaction and conflict between humans and macaques are common in many areas, including the Kosumpee Forest Park (KFP) in Maha Sarakham, Northeast Thailand. *Staphylococcus aureus* is a widespread opportunistic bacterial pathogen that may cause community-acquired, hospital-related, and livestock-associated infections [6]. Methicillin-resistant *S. aureus* (MRSA) is an important antimicrobial resistant opportunistic pathogen, which can colonize humans, domestic pets, livestock, and wildlife [7]. The MRSA is different from methicillin-susceptible *S. aureus* (MSSA) due to the carriage of the *mecA* gene on a variety of distinct mobile genetic elements known as Staphylococcal Cassette Chromosome *mec* (SCC*mec*). The *mecA* gene codes for an alternative penicillin-binding protein [PBP2a] [8, 9].

Recent studies have demonstrated that wild rhesus macaques (*Macaca mulatta*) living around temple areas in the Kathmandu valley in Nepal with frequent human contact, and those in remote areas with limited human contact were colonized with MRSA and/or MSSA [2, 3, 10]. In the first pilot study, saliva samples were collected from 59 *M. mulatta* [10]. Four (6.8%) macaques were positive for MRSA with three isolates of ST22 SCC*mec* type IV. This sequence type (ST) is a pandemic strain found in Asia, Africa, Middle East, and Europe and identified in hospitalized patients in Nepal [10]. Subsequent studies of wild *M. mulatta* found that MRSA isolates were antibiotic resistant and were the same STs as other human MRSA isolates found both within Nepal and other parts of the world [2]. Some of the MSSA isolates from the macaques were also antibiotic resistant and had the same STs as found in humans or animals/livestock MSSA isolates. However, a number of MSSA isolates were not antibiotic resistant and not genetically related to human or other animal isolates, and appeared to be unique to non-human primates [3, 11]. These MSSA isolates were found in both macaques that interacted with people, as well as, those living in remote areas, and were from both *M. mulatta* and *Macaca assamensis* hosts [11]. The data from these studies suggest a transmission of MSSA/MRSA isolates between macaque species and an indication of anthropozoonotic transmission of “human” strains from contaminated environments and human and/or livestock contacts with wild macaques. Other studies have found MSSA isolates in African non-human primates and zoos [12, 13]. Recently, *S. aureus* clonal complex 75 (CC75) has been reclassified as a new species *Staphylococcus argenteus* [14]. The phenotypic characteristics of *S. argenteus* are very similar to *S. aureus*, except that the colonies often display a non-pigmented presence on blood agar due to the lack of genes producing staphyloxanthin [15, 16]. However, approximately 10% of *S. aureus* isolates produce white colonies, resulting in misidentification of *S. argenteus* isolates as the more common *S. aureus* [17, 18]. The routine use of the PCR assay targeting non-ribosomal peptide synthetase (*NRPS*) gene has been used as a rapid method for *S. argenteus* identification[19]. *Staphylococcus argenteus* has been identified in Thailand patients and animals [14, 16], and from non-human primate isolates around the world [20].

Recently, a number of clinical reports of *S. argenteus*, causing a disease similar to that of *S. aureus*, in both humans and animals has increased [21, 22]. This study aimed to investigate the prevalence of *S. aureus* and *S. argenteus* carriage in buccal swab samples from wild long-tailed macaques (*M. fascicularis*) at KFP, Thailand. The isolates were tested using PCR methods for detecting *mecA* to identify MRSA and *NRPS* genes for *S. argenteus* identification.

## Materials and Methods

### Ethical approval

This research was approved and conducted in accordance with the animal use protocol approved by the Institutional Animal Care and Use Committee at Mahasarakham University (MSU) (protocol numbers 0009/2016). All animal samples collection complied with the current laws of Thailand.

### Study period and location

**Figure-1:**
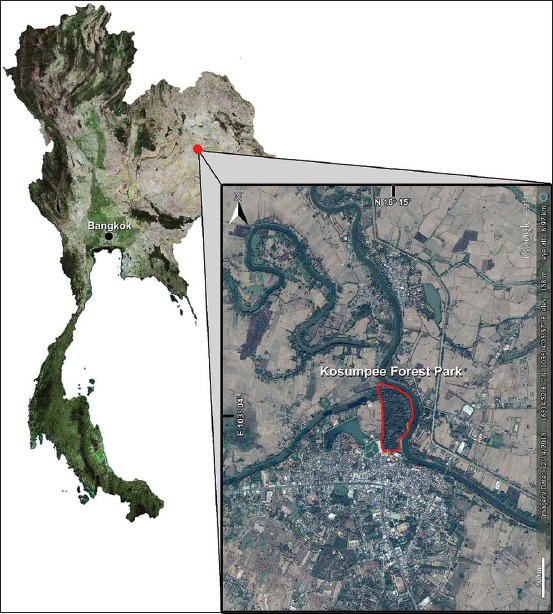
Map of the Kosumpee Forest Park, Kosum Phisai District, Maha Sarakham, Northeast Thailand. The map illustrates the close proximity of the human community to the forest park study site [Source: Randall C. Kyes].

**Figure-2:**
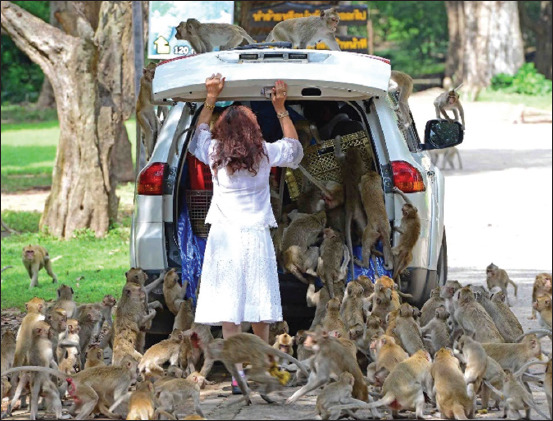
Wild long-tailed macaques at Kosumpee Forest Park have extensive interaction with local residents and tourists [Source: Randall C. Kyes].

The cross-sectional study was conducted during November-2018 at KFP; N16° 15’ 12.6” E103° 04’ 02.0”, Kosum Phisai district, Maha Sarakham, Northeast Thailand (Figure-1). The KFP consists of a mixed deciduous forest of approximately 0.2 km^2^, located along the Chi River and bound by agricultural areas and the town of Kosum Phisai to the south. The KFP is home to a free-ranging population of long-tailed macaques (*M. fascicularis*) of approximately 850 macaques (at the time of the study), distributed among five social groups [23]. The macaques have extensive interaction with local residents and tourists (Figure-2). This can lead to various levels of conflict including aggressive interactions, crop raiding, damage to buildings, as well as, retaliatory responses by the people directed toward the macaques (e.g., throwing rocks/sticks, use of sling shots, etc.) [23, 24].

### Sampling procedures

Buccal samples were collected from 30 randomly selected macaques living in KFP and the surrounding urban area during trapping for health screening checkups. The macaques were captured in a soft nylon mesh cage with a wooden frame (150 × 40 × 40 cm) baited with groundnuts and bananas. Tiletamine-zolazepam (Zoletil^®^ 100 mg/mL, Virbac, Carros, France) was injected intramuscularly to sedate the macaques through 5 mL anesthetic blowpipes after visually assessing a body weight for each macaque in the mesh cage. After anesthesia, the macaques were removed from the cage and vital signs were monitored. Buccal swab samples were then collected aseptically using a sterile buccal swab collection kit with transport media (Deltalab, Barcelona, Spain). All trapping and sampling procedures were conducted by wildlife veterinary specialists from MSU following the protocol approved by the MSU Institutional Animal Care and Use Committee (ethics statement above) and the Thai Department of National Parks. All buccal swab samples were stored at 4°C and taken to the One Health Research Unit, Veterinary Public Health Laboratory, Faculty of Veterinary Sciences at MSU for bacterial testing within 6 h of collection.

### *Staphylococcus aureus* screening

The 30 buccal swabs were cultured on Baird-Parker Agar supplemented with egg yolk tellurite emulsion (Oxoid, Hampshire, UK). Bacterial plates were placed in an incubator at 37°C for 24 h and suspected *S. aureus* colonies were further characterized. Tentative identification of suspected bacterial colonies was performed using Gram-staining, catalase test, tube coagulase test, mannitol salt agar test, deoxyribonuclease test, and Staphaurex™ Plus latex agglutination test (Thermo Fisher Scientific, Massachusetts, USA) [25].

### Antimicrobial susceptibility testing for MRSA

Antimicrobial susceptibility for β-lactam resistance of the isolates was performed by disk susceptibility testing using Mueller-Hinton agar (Oxoid) and cefoxitin disk (30 mg Oxoid). The results were interpreted using the Clinical and Laboratory Standards Institute guidelines M100 to identify methicillin resistance [26]. *Staphylococcus aureus* ATTC^®^25923 was used as a control MSSA strain and *S. aureus* ATCC^®^43300 was used as MRSA control strain for antimicrobial drug susceptibility testing.

### Polymerase chain assay

**Figure-3:**
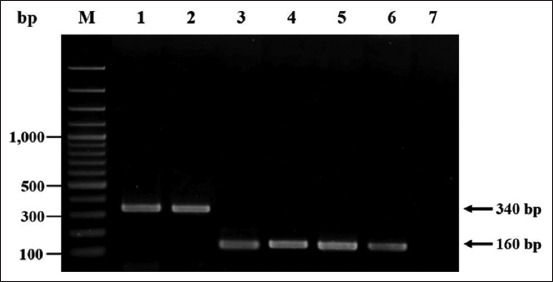
Polymerase chain reaction (PCR) amplicons of non-ribosomal peptide synthetase gene (*NRPS*) of *Staphylococcus aureus* and *Staphylococcus argenteus* isolated from wild long-tailed macaques. Lane M, DNA marker; lanes 1–2, PCR amplicons of isolates number 17.1 (Monkey ID: M17) and 28.4 (Monkey ID: M28) which was suspected as *S. argenteus*, respectively; lanes 3–5, PCR amplicons of isolates number 1.1 (Monkey ID: M1) and 22.1 (Monkey ID: M22) which was suspected as *S. aureus*; lane 6, PCR amplicon of *S. aureus* ATTC 25923; and lane 7, negative control.

The isolates’ DNA was extracted using a DNA genomic extraction kit (Geneaid, Taipei, Taiwan), according to the manufacturer’s instructions. The DNA extraction products were measured by the OD 260/280 nm ratio using a NanoDrop 1000 Spectrophotometer (Thermo Scientific, New Jersey, USA) and kept at −20°C until tested. DNA samples were PCR tested for the presence of the *mecA* gene to confirm MRSA and *NRPS* genes to differentiate *S. argenteus* from *S. aureus*. The PCR reaction mixtures were prepared in a total volume of 25 μL with 10 μmol/L of both forward and reverse primers, KAPA2G™ Robust HotStart ReadyMix PCR Kit (Kapa Biosystems, Wilmington, MA, USA), DNA template, and sterile deionized water. Amplification of the *mecA* gene was performed using specific primers *mecA* F (5′-AAAATCGATGGTAAAGGTTGGC-3′) and *mecA* R (5′- AGTTCTGGAGTACCGGATTTGC-3′). These primers produced a PCR amplicon of 533 bp with the MRSA isolates while and the MSSA isolates were negative [27]. The *NRPS* gene detection was performed by PCR with specific primers (*NPRS*1 5′-TTGARWCGACATTACCAGT-3′ and *NPRS*2 5′-ATWRCRTACATYTCRTTATC-3′) which were derived from a previous study that published these nucleotide sequences [19]. For the analysis of the *NRPS* gene, the PCR products were 160 bp and 340 bp (*S*. *aureus* and *S. argenteus*, respectively) (Figure-3).

PCR amplification was performed using a thermocycler (Biometra GmbH, Jena, Germany) as follows: 4 min initial denaturation at 94°C, 35 cycles of denaturation at 94°C for 30 s, annealing at 55°C for 30 s (*mecA* gene primers) and 53°C for 30 s (*NRPS* gene primers), extension at 72°C for 40 s, and then final extension at 72°C for 10 min. The PCR products were analyzed by 1.5% agarose gels stained with 0.5 mg/mL ethidium bromide in 0.5× TBE buffer and visualized under ultraviolet light. *Staphylococcus aureus* ATCC^®^43300 served as the positive control strain for *mecA* gene. *Staphylococcus aureus* ATTC^®^25923 and ATCC^®^43300 were used as the positive control strains for the *NRPS* gene while distilled water was used as the negative control.

## Results and Discussion

### Prevalence of MSSA/MRSA and *S. argenteus*

Fifteen macaques (50%) were colonized with *Staphylococcus* spp. and 21 isolates were identified (Table-1). Of the 21 isolates, 5 (23.8%) were MSSA, 14 (66.7%) were MRSA, and 2 (9.5%) were *S. argenteus*. Four (27%) of the macaques carried two different isolates each: M1, M21, and M22 carried both a MRSA and MSSA isolates and M28 carried one MRSA and one *S. argenteus* isolate. One animal (M17) carried only a *S. argenteus* isolate (Table-1) and the remaining 10 (67%) macaques carried only *S. aureus* (Table-1). The *NRPS* gene analysis (Figure-3) confirmed that the two isolates were *S. argenteus* with an amplicon size of 340 bp versus 160 bp for *S. aureus* (Table-1). The two *S. argenteus* isolates (number 17.1 and 28.4) were from animals M28 and M17 (Table-1) and represented 9.52% of the staphylococci isolated from the macaques at KFP.

**Table-1:** *Staphylococcus* spp. isolated from wild longtailed macaques.

Monkey ID	Isolate no.	*mecA* gene	*NRPS* gene amplicon size (bp)	Bacterial identification
M 1	1.1	+	160	MRSA
	1.2	-	160	MSSA
M 2	2.2	+	160	MRSA
M 4	4.1	+	160	MRSA
M 7	7.3	+	160	MRSA
M 9	9.1	+	160	MRSA
M 15	15.1	+	160	MRSA
M 16	16.1	+	160	MRSA
M 17	17.1	-	340	*S. argenteus*
M 19	19.2	-	160	MSSA
M 21	21.3	-	160	MSSA
	21.1	+	160	MRSA
M 22	22.1	-	160	MSSA
	22.4	+	160	MRSA
M 24	24.1	+	160	MRSA
M 25	25.1	+	160	MRSA
M 26	26.5	-	160	MSSA
M 27	27.3	+	160	MRSA
M 28	28.1	+	160	MRSA
	28.4	-	340	*S. argenteus*
M 29	29.5	+	160	MRSA

MRSA=Methicillin-resistant *Staphylococcus aureus*, MSSA=Methicillin-susceptible *Staphylococcus aureus*, *S. argenteus*=*Staphylococcus argenteus*

### Comparing the Thailand non-human primate carriage with previous studies

The prevalence of *S. aureus* carriage in this study (50%) was higher than the reported prevalence of 19.4% (six isolates) in other non-human primates including *Colobus guereza, Papio anubis, Trachypithecus francoisi*, and *Rhinopithecus roxellana* (31 samples) from Yangzhou Ecological Zoo in Jiangsu, China [12]. From their study, 1 isolate (16.67%) from *T. francoisi* (fecal sample) was identified as MRSA and 5 isolates (83.33%) were MSSA. The prevalence of *S. aureus* in Thailand is similar to that the reported in laboratory macaques (n=176, 58.7%) from Chicago, USA [28], which included 152 *M. fascicularis* and 148 *M. mulatta*. The carriage rate in this study was 10.8% for MRSA and the remaining 89.2% were identified as MSSA carriers. The results of this study are also similar to the research with *M. mulatta* and *M. assamensis* (n = 40) in Nepal [3, 29].

To the best of our knowledge, this is the first study to report the presence of *S. argenteus* in wild long-tailed macaques from Thailand. To date, *S. argenteus* has been found in both humans and animals from many regions of the world, including Thailand [21, 29]. Although *S. argenteus* has been identified in dairy cows [18, 30], rabbits [31], canine [22], and pigs [32], its prevalence in wildlife is understudied.

## Conclusion

This study is the first to report the prevalence of *S. aureus*, MRSA, and *S. argenteus* from wild non-human primates in Thailand. Although the number of animals tested was limited, the prevalence rate of *S. aureus* and *S. argenteus* carriage was high (50%). The Nepalese macaque isolates could be distinguished based on antibiotic carriage which was associated with these isolates being part of known human and livestock clones, while novel clones did not carry antibiotic resistance genes [2, 3, 11]. Thus, further characterization of the isolates in the current study, including MIC for other antibiotics, as well as, multilocus sequence typing could help provide insight into whether the Thailand isolates are related to previously characterized clones from humans and/or livestock. *Staphylococcus argenteus* carriage has not previously been identified in the Nepalese macaques but two animals from Thailand carried *S. argenteus* in this study.

## Authors’ Contributions

NP, TC, KS, and SP: Conceptualized and designed the study. NP, TC, KS, and SP: Collected the samples. RCK, PT, TT, PK, and AK: Filed sampling coordinator. TC, KS, and SP: Performed the laboratory experiments. NP, TT, PK, PT, and RCK: Supervised the study. NP, TC, KS, and SP: Conducted molecular work. NP, RCK, and MCR: Analyzed the data. NP, RCK, and MCR: Drafted and revised the manuscript. All authors have read and approved the final manuscript.
